# A Strategy for Identifying Quantitative Trait Genes Using Gene Expression Analysis and Causal Analysis

**DOI:** 10.3390/genes8120347

**Published:** 2017-11-27

**Authors:** Akira Ishikawa

**Affiliations:** Laboratory of Animal Genetics and Breeding, Graduate School of Bioagricultural Sciences, Nagoya University, Nagoya 464-8601, Japan; ishikawa@agr.nagoya-u.ac.jp; Tel.: +81-52-789-4101

**Keywords:** QTL, QTG, gene expression, causal analysis

## Abstract

Large numbers of quantitative trait loci (QTL) affecting complex diseases and other quantitative traits have been reported in humans and model animals. However, the genetic architecture of these traits remains elusive due to the difficulty in identifying causal quantitative trait genes (QTGs) for common QTL with relatively small phenotypic effects. A traditional strategy based on techniques such as positional cloning does not always enable identification of a single candidate gene for a QTL of interest because it is difficult to narrow down a target genomic interval of the QTL to a very small interval harboring only one gene. A combination of gene expression analysis and statistical causal analysis can greatly reduce the number of candidate genes. This integrated approach provides causal evidence that one of the candidate genes is a putative QTG for the QTL. Using this approach, I have recently succeeded in identifying a single putative QTG for resistance to obesity in mice. Here, I outline the integration approach and discuss its usefulness using my studies as an example.

## 1. Introduction

Most traits of biological and economic importance, including traits for human complex diseases (e.g., autoimmune, metabolic and psychiatric diseases), traits for agricultural and livestock products (e.g., crop yield, meat quality and egg production) and molecular traits (e.g., gene expression, protein expression, DNA methylation, histone modification and metabolites), are quantitative in nature and are hence called complex or quantitative traits. Quantitative traits are intricately regulated by many genetic loci, referred to as quantitative trait loci (QTL), environmental factors and their interactions. QTL mapping is an unbiased phenotype-driven method that detects statistical associations between genotypes of genetic markers and phenotypic values for a quantitative trait of interest, and it is used to localize QTL affecting the trait to chromosomal regions containing marker loci that are significantly associated with the trait in an attempt to understand the genetic architecture of trait variation. There are two common approaches for QTL mapping. One approach is a genome-wide association study (GWAS) used in outbred populations such as humans and large livestock animals. The other approach is a so-called genome-wide QTL analysis based on linkage analysis in three-generation pedigrees or designed crosses of model animals and small livestock animals such as chickens and pigs [1].

Large numbers of QTL affecting complex disease traits and other quantitative traits have been mapped to almost all chromosomal regions of humans, livestock animals and model animals, and they have been deposited in databases that can be used freely such as the NHGRI-EBI GWAS Catalog [2], the Animal Quantitative Trait Loci Database (Animal QTLdb) [3] and the Mouse Genome Database (MGD) [4]. However, for two main reasons, it remains a great challenge to identify causal quantitative trait genes (QTGs) and further causal genetic variants, called quantitative trait nucleotides (QTNs), for common QTL with relatively small phenotypic effects. First, by a conventional approach using techniques such as positional cloning (see the Appendix A for definition), it is difficult to narrow down a target genomic interval of a QTL to a very small interval ultimately harboring only one gene that is a potential positional candidate QTG for the QTL. For example, genome-wide QTL analysis with a backcross or intercross population in mice generally localizes a QTL to a large genomic interval of 10–50 centimorgan (cM) in length [5], in which hundreds or thousands of genes are usually contained. To reduce the large interval to a level amenable to positional cloning, additional fine mapping is performed using congenic and subcongenic mouse strains with overlapping and non-overlapping introgressed intervals (see Appendix A) [6,7]. However, it is not easy to narrow the large QTL interval down to a very small interval because of low recombination frequencies within the introgressed interval. This is also true for GWAS fine mapping of multiple single nucleotide polymorphisms (SNPs), associated with a phenotype on a linkage disequilibrium interval, to resolve a single candidate QTN [8]. Second, candidate QTNs with small phenotypic effects are frequently found in non-coding regions of the genome, including promoters, introns and transcription factor binding sites, in humans [9] and mice [10].

**Figure 1:**
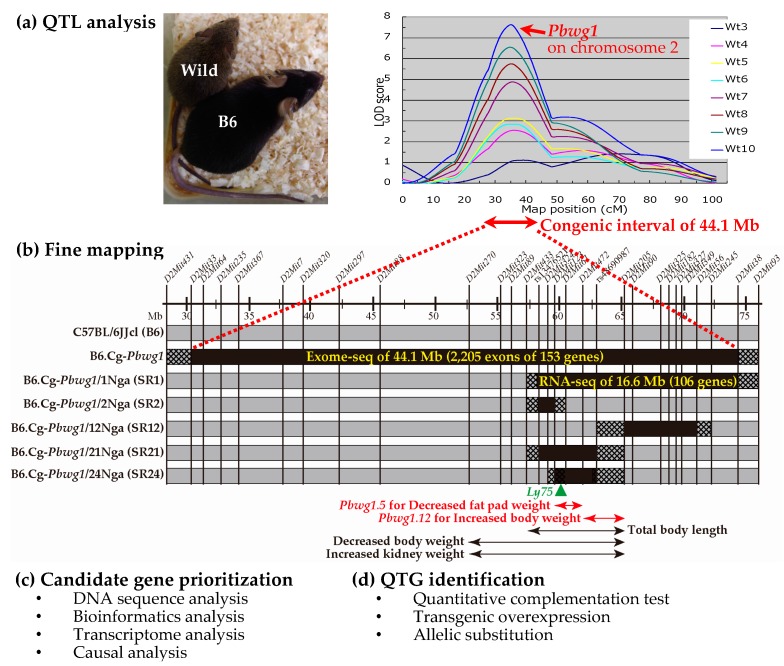
Overview of a strategy from quantitative trait loci (QTL) and quantitative trait genes (QTGs) identification using our studies as an example. (**a**) QTL analysis in an intersubspecific mouse population between wild *Mus musculus castaneus* and the C57BL/6JJcl (B6) inbred strain. The picture shows adult wild and B6 male mice at 20 weeks after birth (photographed by Keita Makino, Graduate School of Bioagricultural Sciences, Nagoya University, Japan). Twenty-four QTL for body weights at 3 weeks (Wt3) to 10 weeks (Wt10) of age are mapped [12,13,14], and among the QTL the most potent QTL (named *Pbwg1*) on mouse chromosome 2 is depicted as logarithm of odds (LOD) score plots. The figure was remade from previous data [14]; (**b**) Fine mapping of *Pbwg1* using the founder congenic strain (B6.Cg-*Pbwg1*) and subsequent subcongenic strains (B6.Cg-*Pbwg1*/Nga#, abbreviation: SR#). The black and grey bars show minimum intervals derived from the wild and B6 mice, respectively. The hatched bar shows an interval where recombination occurred. The map positions (mega base pairs (Mb)) of DNA markers (*D2Mit#* and *rs#*) are approximately shown on the horizontal line. The horizontal double-headed arrows indicate the intervals of QTL for body weight and body composition traits [15,16,18,20], and among the QTL the intervals of *Pbwg1.5* and *Pbwg1.12* are highlighted by red [18]. The effects of the QTL alleles derived from the wild mouse are indicated with the arrows. The green triangle indicates the position of the *Ly75* (lymphocyte antigen 75) gene, a putative QTG for *Pbwg1.5* [19]; (**c**) Candidate gene prioritization using DNA sequence analysis, bioinformatics analysis, transcriptome analysis and causal analysis. In our previous studies, exome-seq analysis of the funder congenic interval [18] and RNA-seq analysis of the SR1 subcongenic interval [19] were performed. Furthermore, bioinformatics analyses (see Table 1) and the causal inference test (see Figure 2) using gene expression data were carried out; (**d**) QTG identification. To identify a QTG, the quantitative complementation test is performed as shown in Figure 3. To validate the QTG, a transgenic overexpression experiment is performed. Furthermore, to identify a QTN within the QTG, allelic substitution experiments using gene editing techniques such as the CRISPR/Cas9 system are performed. See text for details of each analysis.

The laboratory mouse has long been used worldwide as a pilot model animal for elucidating the genetic basis of complex disease traits and quantitative traits in humans and livestock because of its small body size, cost-effective rearing, easy development of knockout and transgenic mice, and large amount of genomic information that is freely available [11]. We previously revealed many QTL affecting postnatal body weight and growth from an untapped resource of wild *Mus musculus castaneus* mice trapped in the Philippines, by genome-wide QTL analysis in an intersubspecific backcross population between the wild *M. m. castaneus* mice and C57BL/6JJcl (B6), a common inbred strain prone to obesity and type-2 diabetes (Figure 1a) [12,13,14]. Fine mapping using congenic and subcongenic strains carrying a major body weight QTL (named *Pbwg1* (postnatal body weight growth 1)) on mouse chromosome 2 revealed two unique QTL affecting body weight (*Pbwg1.12*) [15] and the weight of white fat pads (*Pbwg1.5*) [16]. The wild-derived allele at *Pbwg1.12* increases body weight despite the fact that the wild mouse has a smaller body size than that of B6 (Figure 1a) [15], whereas at *Pbwg1.5* it shows resistance to obesity [17]. Exome sequencing (exome-seq) and bioinformatics analysis revealed two candidate genes for each of *Pbwg1.12* and *Pbwg1.5* [18]. Finally, by using an integrated approach of mRNA expression analysis and causal analysis inferring causal relationships between genotypes, gene expression and trait values, we succeeded in revealing that *Ly75* (lymphocyte antigen 75) is a putative QTG for *Pbwg1.5*, though we did not succeed in finding a QTG for *Pbwg1.12* [19].

In this review, I outline a strategy from QTL to QTG identification using an integrated approach of gene expression analysis and causal analysis. I also discuss the usefulness of the integration strategy using our studies in mice as an example. The strategy can greatly reduce the number of candidate QTGs and it provides statistical evidence that candidate QTG expression causally mediates between genotype and trait variation.

## 2. Quantitative Trait Loci Analysis

In mice, genome-wide QTL analysis is performed in a backcross population or an intercross population obtained from crosses between two inbred strains to map QTL for a given trait to large genomic intervals. It is generally recommended to use an F_2_ intercross population because three possible genotypes for QTL mapped are segregated in the F_2_ population, allowing estimation of the mode of inheritance of the QTL. However, in our QTL analysis, a backcross population was developed as a QTL mapping population because the population was used for another research purpose as well. That is, wild male mice obtained from a cross between a pair of wild-caught *M*. *m*. *castaneus* mice of unknown ages were mated with B6 females to produce F_1_ hybrids, and the F_1_ hybrids obtained were backcrossed to their own wild male parents. Genome-wide QTL analysis in the backcross population obtained revealed 24 QTL for body weight and growth on 13 chromosomes including X chromosomes [12,13,14]. Among the 24 loci, *Pbwg1* on mouse chromosome 2 is the most potent QTL affecting body weight from 3 to 10 weeks after birth (Figure 1a) [13,14].

## 3. Fine Mapping

After QTL analysis, fine mapping is performed using congenic and subcongenic strains in order to (1) confirm the presence of the QTL detected by the initial genome-wide QTL analysis and (2) make the initial large QTL interval as small as possible. In our studies, we firstly developed a congenic strain with a 44.1 mega base pairs (Mb), wild-derived genomic interval carrying *Pbwg1* on the genetic background of the B6 strain (Figure 1b). We further constructed more than 20 subcongenic strains with overlapping and non-overlapping introgressed intervals which together span the entire congenic interval, some of which are shown in Figure 1b as examples. Next, using the congenic and subcongenic strains developed, we took two strategies for fine mapping of *Pbwg1*: a unique strategy that I here call interval-specific QTL analysis for the first time, and a modified method of traditional congenic/subcongenic analysis. In the unique strategy, an F_2_ population of 269 mice was developed by an intercross between the original congenic strain with an approximately 28 cM (44.1 Mb) introgressed interval and the background B6 strain, and then a genetic linkage map for 14 microsatellite markers on mouse chromosome 2 was constructed with an average marker spacing of 1.6 cM and a total length of approximately 20 cM. Interval-specific QTL analysis was performed with the 269 F_2_ mice and the limited linkage map and it revealed that nine QTL, accounting for 4.4–9.6% of total phenotypic variance in body weight and body composition traits, are clustered in the congenic interval [16]. Including our other studies, a total of 12 linked QTL were mapped to the congenic interval [15,16,18,20,21], as some of the loci are shown in Figure 1b. Among the loci, a unique QTL *Pbwg1.5* was found. The wild-derived allele at *Pbwg1.5* decreased gonadal fat pad weight [16] and showed resistance to obesity in mice fed both standard and high-fat diets [17]. Thus, our studies suggested that initially, a large genomic interval of a single QTL identified by genome-wide QTL analysis can be dissected into small intervals containing additional linked QTL for a given trait and related traits. Furthermore, it is considered that, if consomic strains (see Appendix A) are available, then interval-specific QTL analysis can be used as a possible option to fine-map QTL.

In traditional congenic/subcongenic analysis, phenotypic values are compared between homozygous congenic/subcongenic strains and the background strain and/or among homozygous congenic/subcongenic strains. These traditional congenic/subcongenic analyses frequently fail to confirm the phenotypic effect of the QTL. To overcome the failure in traditional congenic/subcongenic analyses, I propose a modified method of the traditional analysis, in which segregating F_2_ populations obtained from intercrosses between each of the subcongenic strains and the background strain are used. The use of the segregating F_2_ animals can randomize environmental effects, such as litter size and micro-rearing conditions, and genetic effects of contaminating donor and recipient alleles on unwanted small genomic regions. Furthermore, it can minimize effects of genetic factors, including maternal genetic effects, genomic imprinting effects, epigenetic effects and other genetic effects, as much as possible because genetically identical F_1_ dams and F_1_ sires are used to produce the F_2_ animals. Using the traditional and modified methods, we were in fact able to find two closely linked QTL (*Pbwg1.11* and *Pbwg1.12*) with opposite effects on body weight. The wild-derived allele at *Pbwg1.11* reduced body weight, whereas at *Pbwg1.12* it surprisingly increased body weight [15], despite the fact that the wild mouse has a smaller body size than that of the B6 mouse (Figure 1a). To further define the genomic interval of two unique QTL (*Pbwg1.5* and *Pbwg1.12*) as small as possible, four different populations of F_2_ mice were produced from intercrosses between the B6 strain and each of four subcongenic strains (SR1, SR2, SR12 and SR21) (Figure 1b). Finally, *Pbwg1.5* and *Pbwg1.12* were fine mapped to a 3.6 Mb interval and the neighboring 2.1-Mb interval, respectively [18], as shown in Figure 1b. These examples indicate that the modified method may work well to dissect an interval of closely linked QTL into different intervals. It is very likely that this success depended on the introgressed intervals of subcongenic strains created.

Alternative approaches, allowing fine mapping of QTL to a few cM intervals through the accumulation of recombination events over many generations, are to use either outbred populations such as advanced intercross lines (AILs) [22] or multi-parental populations such as the heterogeneous stock (HS) [23,24], the collaborative cross (CC) [25] and the diversity outcross (DO) [26]. See the Appendix A for definition of these outbred and multi-parental populations. By QTL analysis in these populations, QTL can be mapped at high resolution. However, the QTL mapped are only loci involved in a gene pool limited to the founder inbred strains of the populations used. For example, whole genome sequencing of 69 CC inbred strains developed from 8 founder inbred strains (see Appendix A) revealed that most of the genomes of the 69 strains is derived from *M*. *m*. *domesticus* subspecies and that the genomic contribution of two wild-derived strains (CAST/EiJ and PWK/PhJ originating in *M*. *m*. *castaneus* and *M*. *m*. *musculus* subspecies, respectively) to the 69 strains is very low [25]. It is unlikely that the genetic variation of the founder strains covers completely vast genetic variation in human diseases that have naturally occurred. If unique animal models derived from different founder strains in origin are used, genes found in the unique animal models will not be always involved in the gene pool of the founder inbred strains of the outbred and multi-parental populations. Hence, researchers will often have to develop original congenic and subcongenic strains to fine map QTL.

## 4. Candidate Gene Prioritization

### 4.1. Strategy

After fine mapping, DNA sequence analysis, bioinformatics analyses, transcriptome analysis, causal analysis and other analyses are used to prioritize candidate genes in a small genomic interval of a fine-mapped QTL (Figure 1c). Since it is becoming clear that a single analysis is not enough to prioritize candidate genes for a QTL, integration of different analyses is generally employed to efficiently find a putative QTG.

In our studies, we employed four analyses, as shown in Figure 1c. First, since DNA sequence data is not available for our wild mouse captured in the Philippines, exome-seq analysis of 153 genes in the 44.1 Mb interval of the original B6.Cg-*Pbwg1* congenic strain was performed and revealed a large number of DNA variants between the wild mouse sequence and the mouse reference sequence (RefSeq mm9) of the C57BL/6J strain. That is, 840 synonymous SNPs (sSNPs), 334 nonsynonymous SNPs (nsSNPs), 9 deletions, 10 insertions and 3 stop codons were found in 2205 exons of 153 genes in the congenic interval. Among these variants, in a 5.9 Mb fine-mapped interval carrying *Pbwg1.5* and *Pbwg1.12*, only sSNPs and nsSNPs were found as shown in Table 1. Alternatively, whole-genome sequencing of two parental strains used for QTL analysis may be performed because (1) it has become cheaper and more easily available than a decade ago, and (2) it provides comprehensive sequence information about coding and non-coding regions in one sequencing. As our exome-seq analysis showed, next-generation DNA sequencing will reveal a huge number of DNA variants between two parental inbred strains used for QTL analysis in coding and non-coding regions of the genome. In fact, when two genetically similar mouse substrains, C57BL/6J and C57BL/6N, were sequenced, they differed by 34 SNPs, 2 indels and 15 structural variants [27]. In addition, in mice, whole-genome assemblies of 16 key inbred strains [10] and high-density SNP maps of multiple common inbred strains [28] are available.

Second, bioinformatics analyses using the information about the nsSNPs detected and genes located in the QTL intervals were performed with three web-based software programs, Endeavour, SIFT and PolyPhen-2, which generate a prioritized list of positional and functional candidate genes. Endeavour prioritizes candidate genes on the basis of similarity to training genes that have already been shown to be involved in regulation of body weight and obesity [29]. PolyPhen-2 predicts possible impact of an amino acid substitution on the structure and function of a protein using straightforward physical and comparative considerations [30]. SIFT predicts tolerated and deleterious substitutions for nsSNPs based on the evolutionary conservation of amino acids within protein families [31]. Endeavour ranked *Ly75* (lymphocyte antigen 75) with nine nsSNPs and *Itgb6* (integrin beta 6) with three nsSNPs as the top two candidate genes for *Pbwg1.5* affecting resistance to obesity, and it ranked *Gcg* (glucagon) with one nsSNP and *Grb14* (growth factor receptor-bound protein 14) with two nsSNPs as the top two candidate genes for *Pbwg1.12* affecting increased body weight. PolyPhen-2 predicted that none of the nsSNPs found in *Ly75*, *Itgb6*, *Gcg* and *Grb14* were harmful to protein functions. SIFT predicted that none of the nsSNPs in the four genes caused possible damage to protein function, whereas it predicted that one nsSNP in *Itgb6* (A>C at the position of 2:60,491,216 leading to Sel302Ala) is harmful to protein function (Table 1).

Third, to find differentially expressed genes in the SR1 subcongenic interval carrying *Pbwg1.5* and *Pbwg1.12* (Figure 1b), RNA-seq analysis was carried out in F_2_ mice obtained from an intercross between SR1 subcongenic and B6 strains, and then real-time PCR analysis was performed to validate the gene expression differences. In the F_2_ mice, three possible diplotypes are segregating in a litter for the subcongenic region. That is, two of the three diplotypes are homozygous for either a haplotype (C) derived from the wild mouse or a haplotype (B) from the B6 mouse. The other is heterozygous for both haplotypes. As summarized in Table 2, in a 5.8 Mb target QTL interval, four and three genes were differentially expressed in the liver and gonadal fat pad, respectively. The expression of *Ly75* and *Fap* (fibroblast activation protein) was upregulated in the liver and/or gonadal fat of mice with the C/C diplotype. According to the results, all of the differentially expressed genes are considered as candidate QTGs for *Pbwg1.5* and *Pbwg1.12*. However, this number of genes would be too large and it would be too laborious to do biological studies using genetically engineered animals.

**Figure 2:**
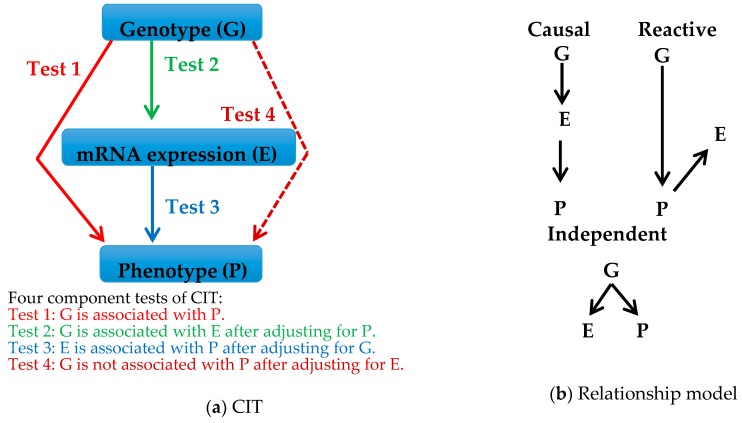
Criteria for the causal inference test (CIT). (**a**) Four component tests of the CIT [32] assessing whether changes in genotype (G) lead to variation in a phenotype (P) through changes in mRNA expression (E); (**b**) Possible relationship models estimated from CIT results. In the causal model, G acts on P through E. In the reactive model, E changes as a result of changes in P. In the independent model, G acts on E and P independently.

Fourth, it can be generally assumed that the genotypic difference in alleles at a given QTL leads to the phenotypic difference in a quantitative trait through changes in gene expression. Causal analysis is used to determine whether a candidate QTG causally mediates between genotype and phenotype. Several methods for causal analysis have been reported [32,33,34,35,36], though the basic statistical concept of conditional dependence is the same in all the methods [37]. In our studies, we performed a causal analysis termed the causal inference test (CIT), which has the simplest statistical principle among the reported methods [32]. CIT analysis is used to assess causal relationships between genotype (G), mRNA expression (E) and phenotype (P), where G is considered as a cause, E is considered as a mediator, and P is considered as the outcome. CIT analysis consists of four component tests that are carried out on the basis of conditional correlation analysis as shown in Figure 2a. Three possible relationship models among G, E and P, i.e., causal, reactive and independent relationships, are obtained as a result of CIT analysis (Figure 2b). In the causal relationship model, G acts on P through E. In the reactive relationship model, E changes as a result of changes in P. In the independent relationship model, G acts on E and P independently. To declare a causal relationship, all four component tests must be satisfied. Researchers search for a gene that fits the causal relationship model. Such a gene must be a putative QTG causally mediating between the genotype and phenotype. There are two advantages of using CIT analysis. One advantage is that the four component tests can filter out genes showing true causal relationships from consequential genes with reactive and independent relationships. The other advantage is that CIT analysis has no priori assumption at the time of testing because the component tests are based on conditional correlation analysis [32]. Thus, CIT analysis can greatly reduce the number of candidate QTGs and it may provide a chance for discovery of novel genes or known genes with unknown functions on the phenotype.

Using 3 genotypes, data for 48 quantitative traits (body weight, body composition and biochemical levels), and data for differentially expressed genes (Table 2) measured in a segregating F_2_ population obtained from an intercross between SR1 subcongenic and B6 strains, CIT analysis was performed [19]. None of the four genes differentially expressed in the gonadal fat pad passed all four CIT component tests. None of the genes differentially expressed in the liver and gonadal fat had causal relationships with body weight and growth traits, suggesting failure to identify a putative QTG for *Pbwg1.12* affecting increased body weight. On the other hand, the CIT provided statistical evidence that only the *Ly75* gene in the liver mediates between genotype and white fat pad weight, suggesting that *Ly75* is a putative QTG for *Pbwg1.5* with a preventive effect on obesity.

The above CIT analysis was performed for the limited number of genes located in a congenic interval. This analysis is generally performed at the genome-wide level. In mice, by CIT analysis and validation studies using genetically engineered animals, *Zfp90* (zinc finger protein 90), *C3ar1* (complement component 3a receptor 1) and *Tgfbr2* (transforming growth factor, beta receptor II) have been identified as new QTGs involved in susceptibility to obesity [33]. In chickens, *LOC770352* (uncharacterized LOC770352), *ADAM10* (ADAM metallopeptidase domain 10) and *C1orf107*/*DIEXF* (digestive organ expansion factor homolog, zebrafish) have been newly reported as putative QTGs underlying anxiety behavior [38]. In humans, causal analysis including the CIT and related methods has been widely used in the past decade to reveal causal epigenetic relationships between gene expression and diseases [39,40,41].

### 4.2. Limitations

As an alternative approach for RNA-seq analysis, genome-wide expression QTL (eQTL) analysis, in which mRNA expression levels of genes are used as quantitative traits, is often performed in the same mapping population as that for the initial genome-wide QTL analysis of a phenotypic trait. The genomic positions of eQTL mapped by genome-wide QTL analysis are compared to those of the QTL for the phenotypic trait. When the positions coincide, the gene for which expression has been detected as the eQTL is considered to be a possible candidate gene for the QTL affecting the phenotypic trait. However, the confidence intervals of multiple eQTL are often overlapped with that of a QTL for the trait. Furthermore, the confidence intervals of the eQTL are not always perfectly matched with that of the trait QTL.

There are two main limitations for CIT analysis. The first limitation is that a mediator that results in showing causality may in fact be acting as a spurious mediator being tightly linked with an unmeasured true causal mediator [32]. The incidence of spurious causal relationships may increase when CIT analysis is performed at the genome-wide level. To overcome this problem, validation studies using genetically engineered animals will eventually be needed. The second limitation is that population stratification, as seen in humans, may lead to spurious causal relationships between genotypes and gene expression and between gene expression and disease [36]. In contrast, generation of population stratification is unlikely in model animals, for which a segregating population such as an F_2_ population has been developed, and hence such a population never shows population stratification. Other minor limitations for CIT analysis are described in detail elsewhere [36,37].

In addition, we failed to find a candidate QTG for the body weight QTL *Pbwg1.12* by CIT analysis. A possible reason for the failure may be that the expression level of *Gcg* was not included in CIT analysis. *Gcg*, a key gene controlling glucose metabolism and homeostasis [42], is prioritized as the top candidate gene (Table 1). However, *Gcg* is mainly expressed in digestive organs such as the pancreas and intestine in mice but not in the liver and fat we examined. It is well known that to choose main organs is an important first step for transcriptome analysis. To overcome this problem and to reduce the costs and increase the efficiency of transcriptome analysis, the use of a public database such as GEO (Gene Expression Omnibus) may be a possible alternative method for obtaining gene expression data. In fact, it is shown that Mendelian randomization analysis can be undertaken using publicly available data [43].

## 5. Quantitative Trait Genes Identification

### 5.1. Strategy

**Figure 3:**
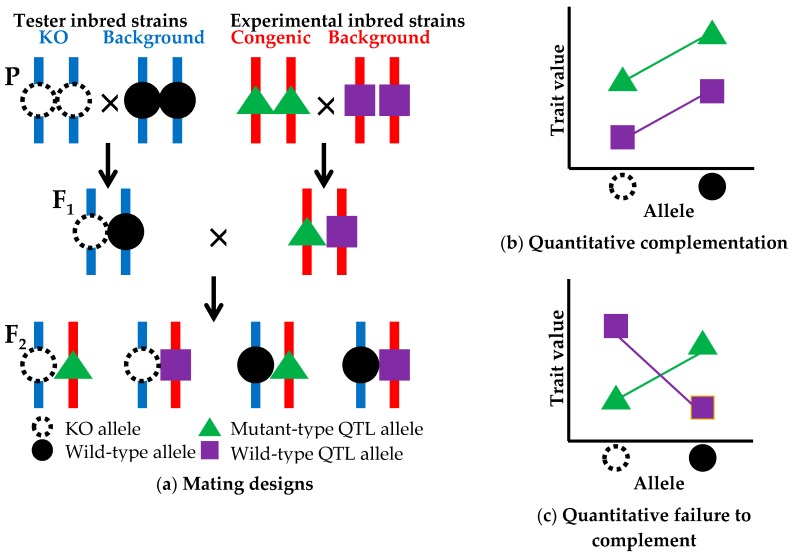
Quantitative complementation test. (**a**) Mating designs using two tester inbred strains (a strain with a knockout (KO) allele (dotted open circle) at a candidate gene locus and its background strain with a wild-type allele (closed circle) at the candidate gene locus and two experimental inbred strains (a congenic strain with a mutant-type allele (green triangle) at a QTL and its background strain with a wild-type allele (purple square) at the QTL. The two tester strains have all the same chromosomes (blue vertical bars) except for the chromosomal position of the KO locus. In the two experimental strains, all chromosomes (red vertical bars) are the same except for the congenic region in which alleles at some loci may be different between the two experimental strains. In the F_2_ animals, four types of genotypes are segregating on a uniform genetic background; (**b**) Quantitative complementation (KO locus ≠ QTL), indicated by no statistical interaction between KO and QTL alleles; (**c**) Quantitative failure to complement (KO locus = QTL), indicated by a significant interaction between KO and QTL alleles.

The above prioritization of candidate genes using CIT analysis reduces the number of candidate genes down to one or a few genes. The next step is QTG identification (Figure 1d), which is established by a quantitative complementation test, or a QTL-knockout interaction test. This complementation test is used to determine whether the genetic locus of the candidate gene is the same as the QTL [44]. Here, I propose a modified method of the complementation test using a common genetic background (Figure 3). In the modified method, two experimental inbred strains, a congenic strain carrying a mutant-type allele at a QTL and its background inbred strain with a wild-type QTL allele, are crossed with each other to produce F_1_ hybrid animals. Likewise, two tester inbred strains, a strain with a knockout (KO) allele at a candidate gene locus within the congenic interval and its background strain with a wild-type allele at the candidate locus, are crossed with each other to produce F_1_ animals. Two types of F_1_ animals obtained from experimental and tester crosses are crossed with each other to produce F_2_ animals. Trait values for the F_2_ animals are measured. There are two advantages for the use of F_2_ animals. One advantage is that all F_2_ animals have the same genetic backgrounds in which all genes are heterozygous except for the congenic region segregating in the F_2_ animals (Figure 3a). The other advantage is that, as described earlier, the use of F_2_ animals whose genotypes are segregating in a litter can randomize environmental effects and contaminating genetic effects of donor and recipient alleles on unwanted chromosomal regions and can minimize the effects of genetic factors such as maternal genetic effects and epigenetic effects as much as possible [18]. In the quantitative complementation test, an interaction effect of the KO allele and the QTL allele on the trait is investigated by a two-way analysis of variance (ANOVA). If the interaction effect is not statistically significant, showing quantitative complementation, then it is interpreted as genetic evidence that the candidate gene locus is not a QTL (Figure 3b). If the interaction effect is significant, showing a quantitative failure to complement, then it can be concluded that the candidate locus is the same as the QTL (Figure 3c), i.e., the candidate gene is a true QTG. However, the possibility that the presence of nearby genes in the congenic interval may result in a spurious significant effect cannot be ruled out. To overcome this problem, biological studies using genetically engineered animals, in which the mutant phenotype is rescued in the congenic strain or is produced in the background strain, will be needed to finally confirm that the gene that has been knocked out is a true QTG. These confirmation studies will be performed together with biological studies for QTN identification as mentioned in a later section.

We are now performing the quantitative complementation test using the SR24 subcongenic strain (Figure 1b), the *Ly75* knockout strain and respective background strains. *Ly75* encodes dendritic and epithelial cells, 205 kDa (DEC-205). *Ly75* knockout mice exhibit abnormalities in CD8-positive T cell morphology and cytotoxic T cell physiology [45]. Hence, it is clear that *Ly75* contributes to immune function. However, the effect of the gene on obesity and related traits has not yet been clarified.

### 5.2. Limitations

By quantitative complementation tests, *Pappa2* (pregnancy-associated plasma protein A2) was previously shown to be as a QTG for a QTL with a small general effect on body size (tail length, bone length and body weight) in mice. However, an interaction effect between *Pappa2* and QTL genotypes was only significant for tail length and body weight at three weeks of age, whereas it was not significant for skull lengths, long bone lengths and body weights at six and 10 weeks [46]. That report indicates that even if a gene that has been knocked out is a QTG for a QTL with a small effect, the results of quantitative complementation tests would not always reach levels of statistical significance. One solution for this problem is to use a simple transgenic overexpression of a candidate gene, which has recently been shown to be efficient for positional cloning of a tail suspension QTL [47] and an adiposity QTL [48] in mice.

## 6. Future Perspective

The number of QTGs identified will continue to increase by use of the approach proposed here, which is backed by several consistent and systematic experimental analyses being performed on a common genetic background controlled by analysis. Most of the QTGs identified would be for QTL having the main effects on phenotypic traits, each of which independently exerts its effect on the phenotypic value. Such QTL can be easily identified by QTL analysis and further development of congenic strains carrying the QTL is straightforward as described in the Appendix A. Recently, it is becoming clear that QTL with epistatic interaction effects on phenotypes make an important contribution to quantitative variation. To identify QTGs for such epistatic QTL remains challenging because of the difficulty of fine mapping the epistatic QTL by congenic/subcongenic analysis. This is a probable limitation for the present approach.

The ultimate goal is to identify a QTN underlying the phenotypic difference in a quantitative trait and to further determine the biological mechanisms linking the genotype and phenotype. The most rigorous proof of QTN identification is to show evidence for an allelic substitution effect of a candidate QTN on the phenotype by replacing the allele of the candidate QTN in one strain with the allele in another strain and vice versa in the same genetic background (Figure 1d). Such an allelic substitution study has now become possible in model animals using the CRISPR/Cas9 system.

Very recently, a 19 bp indel polymorphism in *Rffl*-*lnc1*, a novel predicted long non-coding RNA gene, has been proven to be a QTN for blood pressure and cardiac QT-interval in rats [49]. That study is the first study in mammals in which allelic substitution experiments were performed using the CRISPR/Cas9 system. In that study, targeted rats with deletions of sequences including the 19 bp target segment from the *Rffl*-*lnc1* gene of the wild-type hypertensive Dahl salt-sensitive (S) strain, which has the 19 bp segment, exhibited elevated blood pressures and shorter QT-intervals than those in the wild-type S rats. In contrast, knock-in rats with insertion of the 19 bp segment into the *Rffl*-*lnc1* gene of the S.LEW congenic strain, carrying a blood pressure QTL allele on rat chromosome 10 derived from the normotensive Lewis (LEW) strain without the 19 bp segment, exhibited lower blood pressures and longer QT-intervals than those in wild-type S.LEW congenic rats. These phenotypes of the knock-in rats in the S.LEW congenic strain were very similar to those in the wild-type S rats. In the near future, further advances in genetic engineering technologies such as the CRISPR/Cas9 system will make allelic substitution study more feasible in any mammal, leading to an increase in the number of QTN identifications.

In conclusion, our results in mice may be translatable to human obesity research to prevent obesity, a major health concern worldwide, which is an important predisposing factor for metabolic syndrome. In addition, the results may be translatable to animal breeding programs to produce healthy livestock products.

## Figures and Tables

**Table 1:** Numbers of synonymous single nucleotide polymorphisms SNPs (sSNPs) and nonsynonymous SNPs (nsSNPs) detected by exome-seq analysis of 23 genes in a 2.1 Mb interval of *Pbwg1.5* and the neighboring 3.8 Mb interval of *Pbwg1.12*, ranking of the genes, and damage of protein functions caused by the nsSNPs.

QTL	Gene	sSNP	nsSNP	Gene Ranking ^1^	Damage of Protein ^2^	
					PolyPhen-2	SIFT
*Pbwg1.5*	*Dapl1*	1	0			
	*Tanc1*	21	4			
	*Wdsub1*	6	1			
	*Baz2b*	15	6			
	*March7*	6	0			
	*Cd302*	1	0			
	*Ly75*	27	9	1	Benign	Tolerated
	*Pla2r1*	18	8			
	*Itgb6*	11	3	2	Benign	Affected
	*Rbms1*	2	0			
	*Tank*	1	5			
	*Psmd14*	1	0			
*Pbwg1.12*	*Tbr1*	2	1			
	*Slc4a10*	6	0			
	*Dpp4*	6	0			
	*Gcg*	0	1	1	Benign	Tolerated
	*Fap*	2	2			
	*Ifih1*	17	5			
	*Gca*	3	1			
	*Kcnh7*	6	1			
	*Fign*	4	1			
	*Grb14*	7	2	2	Benign	Tolerated
	*Cobll1*	14	18			

The data are modified from [18]. ^1^ The top two genes were prioritized as candidate genes for each of the two QTL by Endeavour [29]; ^2^ The damage caused by nsSNPs was investigated for the ranked genes by PolyPhen-2 [30] and SIFT [31].

**Table 2:** Differentially expressed genes in a 5.8 Mb genomic interval harboring *Pbwg1.5* and *Pbwg1.12* detected by RNA-seq analysis followed by real-time PCR analysis.

Organ	Gene	Relative Expression Level ^1^			Differences ^2^
		B/B	B/C	C/C	
Liver	*Ly75*	1.00	1.81	3.19	C/C>B/C>B/B
	*Pla2r1*	1.00	−1.58	0.58	B/B≥C/C≥B/C
	*Fap*	1.00	5.89	8.03	C/C>B/C>B/B
	*Gca*	1.00	0.79	0.34	B/B≥B/C≥C/C
Gonadal fat	*Fap*	1.00	1.43	2.11	C/C>B/C>B/B
	*Ifih1*	1.00	−0.47	−0.53	B/B>B/C≥C/C
	*Grb14*	1.00	0.73	0.50	B/B≥B/C≥C/C

The data are modified from [19]. ^1^ The relative gene expression levels were investigated in segregating F_2_ mice with three diplotypes (B/B, B/C and C/C) and are shown as a ratio to B/B. B and C denotes haplotypes derived from B6 and wild mice, respectively; ^2^ Significantly different between the diplotypes at *p* < 0.05.

## Appendix A

Advanced intercross lines (AILs): Lines of mice, rats and other animals developed from an F_2_ intercross population between two founder inbred strains. Animals at the following generations are sequentially produced by randomly intercrossing of animals at the previous generation. The breeding population size of AILs at each generation theoretically requires an effective number of 100 and more animals.

Collaborative cross (CC): A large panel of recombinant inbred (RI) strains of mice (more than 1,000 lines) derived from eight founder inbred strains (A/J, C57BL/6J, 129S1SvImJ, NOD/LtJ, NZO/HILtJ, CAST/EiJ, PWK/PhJ, and WSB/EiJ) representing the three major *Mus musculus* subspecies, *M. m. domesticus*, *M. m. musculus* and *M. m. castaneus*. CAST/EiJ, PWK/PhJ, and WSB/EiJ are derived from wild mice of *M. m. castaneus* in Thailand, *M. m. musculus* in Lhotka, Czech Republic and *M. m. domesticus* in Eastern Shore, MD, USA, respectively. The other five strains are classical strains mainly originating in *M. m. domesticus*. Detailed genome architectures of the eight founder strains can be seen by the Mouse Phylogeny Viewer (MPV), a custom genome browser (http://msub.csbio.unc.edu).

Congenic strain: An inbred strain of animals traditionally developed by repeated backcrossing of animals with a specific genomic interval carrying a QTL from a donor strain to an inbred recipient strain. At each backcross generation, only offspring with the donor interval, which are usually monitored by genetic markers such as microsatellite and SNP markers flanking to the QTL (called marker-assisted selection), are selected for further backcrossing to statistically reduce unwanted donor intervals by 50% per generation. Finally, 10 to 12 generations of backcrossing are required to achieve the congenic strain that theoretically carries approximately 99.9% recipient genome. After that, the congenic strain achieved is maintained by brother-sister mating. This traditional method of congenic production takes approximately three years to complete. Alternatively, the speed congenic method is used. In the method, a congenic strain can be produced in as little as five backcrossing generations through monitoring the introgressed donor interval and genetic background of offspring at each generation by genetic markers. This speed congenic method can establish a congenic strain within two years.

Consomic (chromosome substitution) strains: A panel of special type of congenic strains, produced by using the same method as that of congenic strains described above. Each of the consomic strains has a distinct entire chromosome from an inbred donor strain on the genetic background of an inbred recipient strain.

Diversity outcross (DO): An outbred population developed by random mating of randomly chosen, partially inbred CC mice (see above) at 4-12 generations of inbreeding.

Heterogeneous stock (HS): A stock produced from an eight-way cross of divergent founder inbred strains in mice and rats and then by random mating in a way that minimizes inbreeding.

Positional cloning: A method for identifying a causal gene governing a specific phenotype only by the genomic location of the gene. Linkage analysis in three-generation pedigrees or crosses initially defines a broad genomic region containing the causal gene and other nonrelated genes. To positionally eliminate the nonrelated genes, this broad region is repeatedly narrowed down by genetic analysis using congenic and subcongenic strains (see below) until achieving a very small region ultimately carrying only the causal gene associated with the specific phenotype.

Quantitative complementation test: An allelism test extended a classical complementation test for Mendelian traits to quantitative traits. In the classical complementation test, if two recessive mutant genes are allelic, F_1_ hybrids obtained from a cross between two mutant animals display a mutant phenotype, i.e., the two genes fail to complement each other in the F_1_ hybrids. If the F_1_ show a wild-type phenotype, the two mutant genes are not allelic and complement each other. Likewise, a quantitative complementation test is used to determine quantitatively whether the locus of a candidate gene is the same as a QTL using a knockout (KO) strain for the candidate gene. In the present review, a modified method is proposed to perform the quantitative complementation test on a uniform genetic background, in which two experimental inbred strains of a congenic strain carrying a QTL and its background strain and two tester inbred strains of a KO strain and its background strain are used to obtain two types of F_1_ animals from experimental and tester crosses. An interaction effect of the KO and the QTL allele on a phenotype is investigated by a two-way analysis of variance (ANOVA). See the text and Figure 3 for details.

Subcongenic strain: An inbred strain developed by marker-assisted selection of recombinant animals obtained from an intercross or a backcross between a founder congenic strain with a donor interval carrying a QTL and its background recipient inbred strain. Multiple subcongenic strains are usually developed and have small overlapping and non-overlapping intervals which together span the entire congenic interval. When a panel of subcongenic strains with small intervals partially overlapping by 1 cM step, called interval-specific congenic strains (ISCS), is created, the QTL can be confidently fine-mapped to an interval of 1-2 cM.
